# Oral mucosal injury caused by mammalian target of rapamycin inhibitors: emerging perspectives on pathobiology and impact on clinical practice

**DOI:** 10.1002/cam4.761

**Published:** 2016-06-23

**Authors:** Douglas E. Peterson, Joyce A. O'Shaughnessy, Hope S. Rugo, Sharon Elad, Mark M. Schubert, Chi T. Viet, Cynthia Campbell‐Baird, Jan Hronek, Virginia Seery, Josephine Divers, John Glaspy, Brian L. Schmidt, Timothy F. Meiller

**Affiliations:** ^1^ School of Dental Medicine and Neag Comprehensive Cancer CenterUConn HealthFarmingtonConnecticut; ^2^ Texas Oncology‐Baylor Charles A. Sammons Cancer CenterDallasTexas; ^3^ UCSF Helen Diller Family Comprehensive Cancer CenterSan FranciscoCalifornia; ^4^ Eastman Institute for Oral Health, University of Rochester Medical CenterRochesterNew York; ^5^ Wilmot Cancer Center, University of Rochester Medical CenterRochesterNew York; ^6^ School of Dentistry, University of Washington and Seattle Cancer Care AllianceSeattleWashington; ^7^ New York University College of DentistryNew YorkNew York; ^8^ Penn State Hershey Medical CenterHersheyPennsylvania; ^9^ Tennessee Oncology/Sarah Cannon Research InstituteNashvilleTennessee; ^10^ Beth Israel Deaconess Medical CenterBostonMassachusetts; ^11^ Jonsson Comprehensive Cancer Center, UCLALos AngelesCalifornia; ^12^ School of Dentistry and the Marlene and Stewart Greenebaum Cancer CenterUniversity of MarylandBaltimoreMaryland

**Keywords:** mTOR inhibitor, oral mucosal injury, oral mucositis, stomatitis

## Abstract

In recent years oral mucosal injury has been increasingly recognized as an important toxicity associated with mammalian target of rapamycin (mTOR) inhibitors, including in patients with breast cancer who are receiving everolimus. This review addresses the state‐of‐the‐science regarding mTOR inhibitor‐associated stomatitis (mIAS), and delineates its clinical characteristics and management. Given the clinically impactful pain associated with mIAS, this review also specifically highlights new research focusing on the study of the molecular basis of pain. The incidence of mIAS varies widely (2–78%). As reported across multiple mTOR inhibitor clinical trials, grade 3/4 toxicity occurs in up to 9% of patients. Managing mTOR‐associated oral lesions with topical oral, intralesional, and/or systemic steroids can be beneficial, in contrast to the lack of evidence supporting steroid treatment of oral mucositis caused by high‐dose chemotherapy or radiation. However, steroid management is not uniformly efficacious in all patients receiving mTOR inhibitors. Furthermore, technology does not presently exist to permit clinicians to predict *a priori* which of their patients will develop these lesions. There thus remains a strategic need to define the pathobiology of mIAS, the molecular basis of pain, and risk prediction relative to development of the clinical lesion. This knowledge could lead to novel future interventions designed to more effectively prevent mIAS and improve pain management if clinically significant mIAS lesions develop.

## Introduction

The pathogenesis and clinical phenotype of oral mucositis caused by high‐dose chemotherapy or radiotherapy are well described in the literature 1, 2, 3, 4, 5, 6, 7, 8. In contrast, a unique manifestation of oral mucosal injury has been documented within the past 5 years in association with mammalian target of rapamycin (mTOR) inhibitors 8, 9, 10. This latter toxicity has now emerged as one of the most common adverse events associated with targeted cancer therapies 11, 12, 13, 14, 15, 16, including in women being treated for invasive breast cancer 17, 18.

The clinical trajectory and response to therapy of these two types of oral mucosal injury are distinctly different. It is thus biologically plausible to theorize that there are key pathobiological differences between the two conditions as well; in this context, terminology that appropriately differenciates between the two conditions becomes important. To distinguish between the two types of lesions at the research and clinical levels 7, 19, *mTORI‐associated stomatitis* (mIAS) 9, 10 has become the preferred descriptor of the mTOR inhibitor−associated toxicity.

This review summarizes the state‐of‐the‐science regarding the pathobiology, clinical characteristics, and management of mIAS, and delineates new research directions with an emphasis on the pathogenesis of oral mucosal pain. Additionally, this article is designed to provide the clinician with current management approaches and encourage novel basic, translational, and clinical studies that could enhance the future care of patients with cancer who will receive mTOR inhibitors.

## Phenotype, Incidence, and Pathobiology of mTOR Inhibitor–Associated Stomatitis

**Figure 1:**
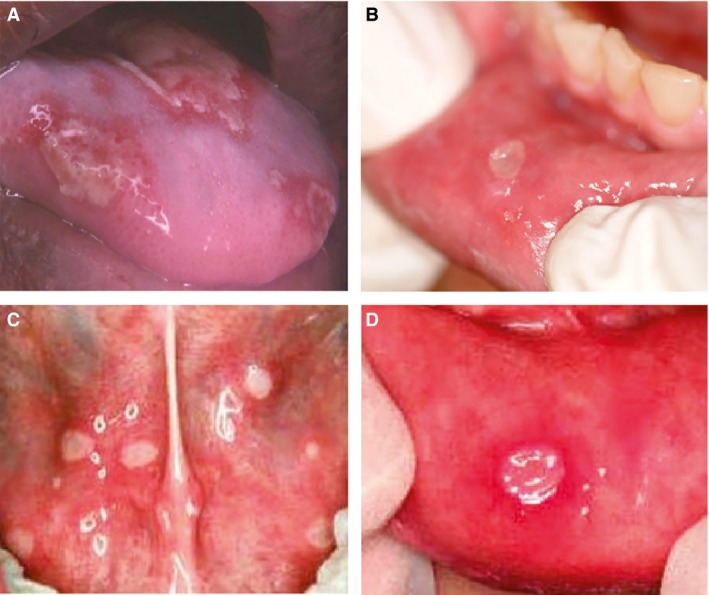
Distinguishing oral mucosal injury of mammalian target of rapamycin inhibitor–associated stomatitis (mIAS) from chemotherapy‐associated oral mucositis, herpetiform stomatitis, and recurrent aphthous ulceration. (A) Conventional chemotherapy‐induced oral mucositis in a 62‐year‐old male with multiple myeloma receiving high‐dose melphalan during peripheral blood stem cell transplant. (B) mIAS in a 58‐year‐old female with breast cancer at ~22 days since receiving everolimus 10 mg/day (note the clinical similarity to solitary herpetiform and recurrent aphthous ulcers with lack of intense inflammatory halo). (C) Herpetiform stomatitis in a 34‐year‐old female in otherwise excellent health. (D) Recurrent aphthous ulceration in an 18‐year‐old male without cancer, with a spontaneous recurrent oral lesion history of approximately three events per year.

mIAS typically presents as multiple or singular round to ovoid ulcerations with regular borders 7. The lesions are commonly less than 0.5 cm in diameter in size and nearly exclusively involve the nonkeratinized oral mucosa (i.e., tongue, floor of the mouth, and labial or buccal mucosa) 7 (Fig. 1). The occurrence of mIAS appears to be dose‐related; the pain and resultant limitations in oral function can be greater than what might be anticipated by the clinician based on the relatively small size of the lesions as compared to other types of oral mucosal injury 9. The intensity of a patient's subjective oral pain experience with mIAS lesions is thus not always commensurate with the degree of oral erythema or ulceration observed clinically.

Incidence of the oral lesions can be high. For example, Martins and colleagues analyzed multiple clinical studies of mIAS in 2,822 patients with cancer who were treated with temsirolimus, everolimus, or ridaforolimus and reported an all‐grade mIAS incidence of 52.9%, with incidence varying among the agents 9. Based on evaluation of clinical trials, the incidence of all grades of stomatitis caused by mTOR inhibitors can vary considerably, ranging from 2% to 78% 9, 20, 21, 22 (Table 1).

**Table 1:** Prevalence of oral mucosal lesions associated with mammalian target of rapamycin inhibitors 9, 20, 21, 22

	Oral mucosal lesion prevalence	
mTOR Inhibitor	All grade	Grade 3/4
Everolimus 20	44–78%^a^	4–9%^a^
Temsirolimus 21	41%^b^	3%^b^
Ridaforolimus 9	54.6%^c^	8.2%^c^
Sirolimus 22	2–10%^d^	0–2%

mTOR, mammalian target of rapamycin.

a Clinical trial experience across all oncology indications; includes mouth ulcers, stomatitis, and oral mucositis.

b Categorized with the preferred term *mucositis* and includes aphthous stomatitis, glossitis, mouth ulceration, mucositis, and stomatitis.

c Data based on five clinical studies involving 194 patients receiving ridaforolimus in an oncology setting.

d Data based on a phase I dose‐escalation study of daily oral sirolimus with weekly intravenous vinblastine in pediatric patients with advanced solid tumors.

**Figure 2:**
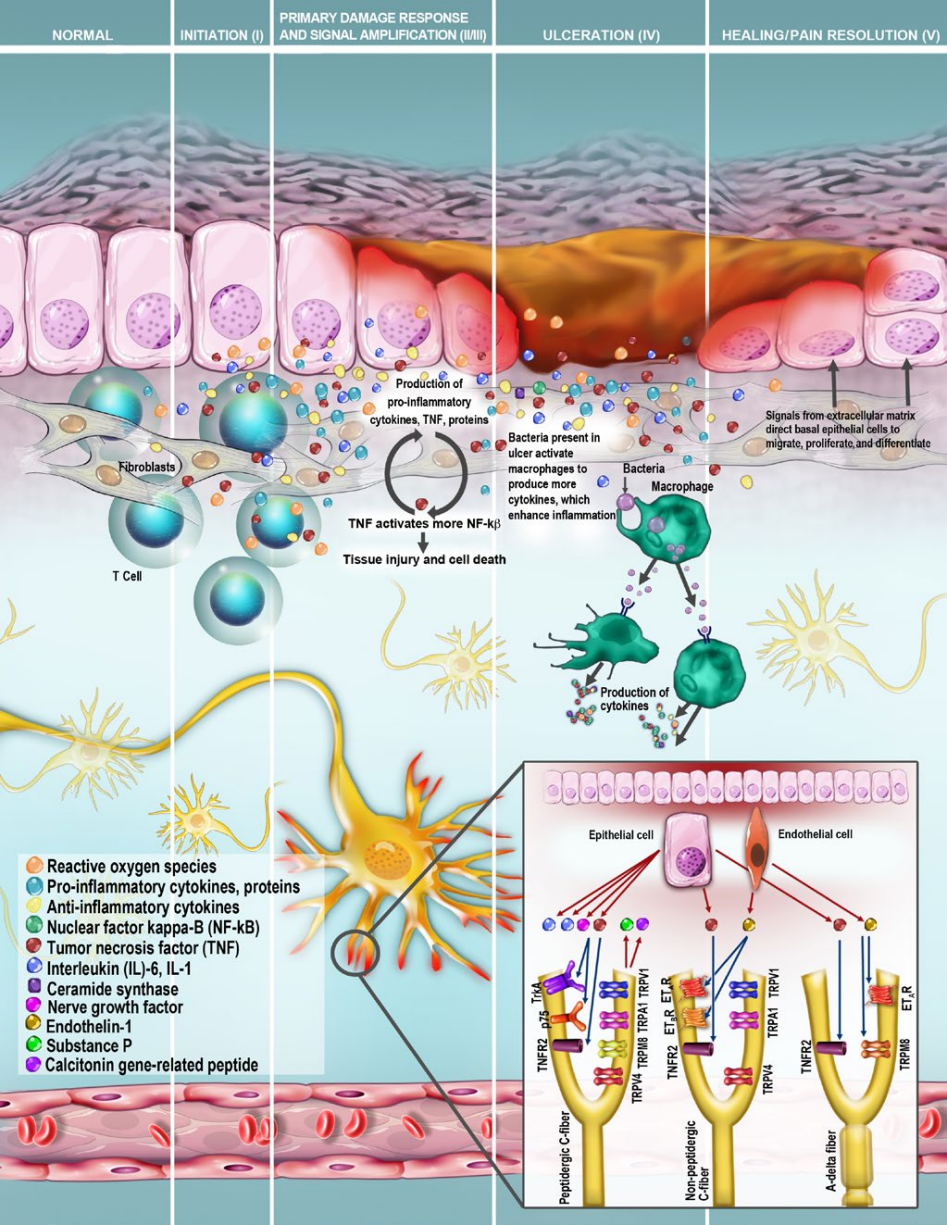
Integration of molecular pain modeling with current pathobiology for oral mucosal injury associated with cancer treatment. The five stages of inflammation in oral mucositis pathogenesis as adapted from the model originally created by Sonis 62. The insert illustrates the integration of the molecular neuropathology of pain into this conceptual framework, with identification of mediators, receptors, and specific nociceptor fiber types within the trigeminal system that likely convey nociception in oral mucositis 40. Transient receptor potential (TRP) receptors associated with mechanical hyperalgesia include the TRPV1 proton receptor, the TRPA1 cold and chemical irritant receptor, the TRPM8 menthol receptor, and the TRPV4 osmolarity receptor. Epithelial cells within the oral mucositis microenvironment secrete interleukin (IL)‐1, IL‐6, tumor necrosis factor (TNF)‐*α*, and nerve growth factor (NGF), triggering an inflammatory cascade. TNF‐*α* activates TNFR2, producing a nociceptive response. NGF binds to either the low‐affinity p75 receptor or the high‐affinity TrkA receptor on peptidergic neurons, in turn modulating neurogenic inflammation. Both C fibers and A‐δ fibers secrete substance P (SP) and calcitonin gene‐related peptide (CGRP) in the periphery, and SP, CGRP, and glutamate in the nucleus caudalis, to mediate nociception. Secretion of endothelin‐1 (ET‐1) within the oral mucositis microenvironment is hypothesized; ET‐1 production is induced by the transcription factor NF‐κB, which is upregulated in oral mucositis. TNFR2 and TRPV4 have not been localized to specific fiber types, and are shown here on multiple fiber types. Adapted with permission from Sonis 62. Molecular pain component of figure adapted with permission from Viet et al. 40. © International & American Associations for Dental Research. Reprinted by Permission of SAGE Publications.

Despite the advances relative to the clinical assessment and treatment of these lesions, delineation of the pathobiology of mIAS remains limited. This contrasts with oral mucositis caused by conventional high‐dose chemotherapy and for which the pathobiology has been studied for the past two decades (Fig. 2) 2, 6, 23, 24, 25, 26, 27. Insights into the mechanism of action of mTOR inhibitors and naturally occurring oral mucosal lesions such as recurrent aphthous ulceration may thus be valuable in informing future research directions involving mIAS.

It is, for example, well established that the mTOR signaling pathway physiologically functions as a central modulator of extracellular and intracellular signaling of mediators and growth factors that in turn regulate molecular and cellular events involved in growth, translation, and metabolism 28. In comparison, mTOR inhibition can cause dysregulation of these molecular and cellular events and include a decrease in expression of CD4+ CD25+ regulatory T cells and T‐cell (predominately CD8 cytotoxic T cells) infiltration and upregulation of heat shock protein 27 and interleukin‐10 7, 29.

Until further research determines the molecular basis of mIAS, it is thus biologically plausible to conceptualize a pathobiologic model of mIAS in relation to the multifactorial mechanistic basis and clinical profile of recurrent aphthous ulceration as defined in the literature (Table 2) 7, 9, 10, 30, 31, 32, 33, 34, 35, 36, 37. Possible risk factors for recurrent aphthous ulceration have classically included anxiety and stress, hormonal alteration, and/or nutritional deficiency.

**Table 2:** Comparison of recurrent aphthous ulceration with mammalian target of rapamycin inhibitor–associated stomatitis 7, 9, 10, 30, 31, 32, 33, 34, 35, 36, 37

Epidemiology and pathobiology: current gaps		
Domain	Recurrent aphthous ulcers	mIAS
Recently identified potential risk factors	Oral microbiotaGenetic governance	UnknownUnknown
Molecular basis	Immune dysregulation, including: Decreased expression of CD4 + CD25+ regulatory T cells Cytotoxic T‐cell infiltration Upregulation of proinflammatory cytokines (e.g., TNF, IL‐2, IL‐6)	UnknownUnknownUnknownUnknown

Clinical characteristics: current similarities		
Domain	Recurrent aphthous ulcers	mIAS
Duration	Typically 7–10 days	<1 week to ≥2 weeks
Clinical phenotype	Shallow ulcerations, with intense erythematous margins Involves nonkeratinized oral mucosa pain	Comparable to recurrent aphthous ulcers
Response to therapeutics	Responsive to locally or systemically administered steroids	Comparable to recurrent aphthous ulcers
Pain management	Steroid management may be supplemented with topical administration of local anesthetics, often delivered via a mucoadherent vehicle	Comparable to recurrent aphthous ulcers

IL2, interleukin 2; IL6, interleukin 6; TNF, tumor necrosis factor.

However, the collective supporting evidence associated with these possible risks remains weak 38. Two recently delineated recurrent aphthous ulceration–related factors described below in the Oral microbiota and Genetic governance sections, provide important context for future studies of mIAS.

### Oral microbiota

Study of the oral microbiota of patients with and without recurrent aphthous ulceration has suggested a relationship between a disturbance in the normal oral mucosal flora and the development of recurrent aphthous ulceration 30. The study of microbiota of the buccal mucosa, rather than of the ulcer *per se*, was a unique design consideration of this analysis. The authors' rationale for this approach was based on the relevance of studying preconditions for recurrent aphthous ulceration development, rather than examining the effect of inflammation directly associated with currently existent ulcerative lesions. The microbiota of noninflamed buccal mucosa differed between patients and controls. The differences were most evident when patients exhibited recurrent aphthous ulceration present during the sampling. These findings raise the question of whether active recurrent aphthous ulceration lesions could alter the microbiota, or whether changes in the normal oral microbiota could induce development of active recurrent aphthous ulceration. Of note is that abundant *Bacteroidales* species at the time of acute recurrent aphthous ulceration may be important in the pathogenesis of this chronic condition 31. These recent findings that delineate a potential association between the oral microbiome and the development of recurrent aphthous ulceration provide an important basis for the continued study of recurrent aphthous ulceration and mIAS.

### Genetic governance

Multiple studies strongly support a genetic basis for recurrent aphthous ulceration. It has been suggested by Slebioda and colleagues that inheritance of specific gene polymorphism coding for proinflammatory cytokine production may be an important contributor to the development of recurrent aphthous ulceration 35. These authors also identified a correlation between the serotonin transcriptase encoding gene polymorphisms and recurrent aphthous ulceration, implicating the potential role of stress and psychogenic stimuli in ulcer development 35. Karasneh and colleagues recently demonstrated the likely relationship between toll‐like receptor 4 rs10759931 polymorphism and recurrent aphthous ulceration 36. They note that future studies would be valuable relative to potentially targeting the toll‐like receptor as a treatment strategy for the lesion 36.

The collective evidence involving the mechanisms of mTOR inhibitors and the lessons learned at the clinical and research levels for recurrent aphthous ulceration may provide important context for new research directed to mIAS pathobiology. This new knowledge could over time also lead to an enhanced ability of the clinician to predict risk for development of mIAS and predict the response to therapeutic intervention on an individual patient‐by‐patient approach.

## Molecular Basis of Oral Mucosal Injury

As noted previously, clinically significant oral pain is a prominent feature of oral mucosal injury caused by conventional and targeted cancer therapies, despite the likely differences in the pathobiology of both conditions. Oral pain can profoundly impact the patient's oral function and overall quality of life (QOL) 39. In addition, the oral pain can limit administration of chemotherapy and mTOR inhibitors, resulting in delivery of suboptimal cancer treatment dosing 3. Unfortunately, treatments with nonopioid analgesics may not adequately control oral pain in some patients 39. These patients may thus require high‐dose opioid therapy, with its attendant toxicities, for control of oral pain.

Despite the clinical importance of pain from cancer therapy‐induced oral mucosal injury, its etiology and pathobiology have not been well defined in the setting of mTOR inhibitors because of difficulty in generating preclinical models that replicate oral ulceration and pain in patients receiving these targeted therapeutics. Drawing on related research may thus provide important insights for future research and management regarding the mIAS pain component.

The model delineated by Viet and colleagues is thus highly relevant in this regard 40. Integration of this pain model into the current oral mucositis pathobiologic paradigm is depicted in Figure 2. The potential translation of this pain modeling with oral mucositis caused by chemotherapy and/or radiation to mIAS may represent a new frontier in the research of pain associated with mIAS. In the Viet model, nociceptive afferents for pain are primarily conveyed by the A‐δ and C fibers in the oral cavity by interaction with varying receptors, including the transient receptor potential (TRP) family of sensory ion‐channel proteins, endothelin‐1 (ET‐1), tumor necrosis factor alpha (TNF‐*α*), and nerve growth factor (NGF) receptors 40. TRP receptors are expressed on trigeminal ganglion neurons involved in thermal and mechanical nociception in the orofacial region 40. Activation of TRP leads to the release of ET‐1, which is a vasoactive and nociceptive peptide that mediates nociception in all trigeminal branches. ET‐1 is implicated in the molecular pathogenesis of oral mucositis though a putative link with the NF‐ĸB transcription factor that is activated in response to oxidative stress 40. Neurotrophic factors such as NGF are secreted by neurons, inflammatory cells, and cancer cells, in turn, mediating pain by the binding of these factors to receptors on peptidergic C fibers 40. Peripheral release from peptidergic C fibers produces the neurogenic inflammation that characterizes the complex pain response involved with oral mucositis 40, 41. Peripheral afferent nociceptors are also sensitized by proinflammatory cytokines, including TNF‐*α*, IL‐2, IL‐6, and IL‐1*β*, generated at the site of oral mucosal injury 40, 41. The effect of anti‐inflammatory cytokines in reducing oral mucositis has been explored. However, although multiple preclinical studies suggested potential benefit, these benefits have not been verified in clinical studies 42.

Further research relative to oral pain associated with oral mucositis caused by chemotherapy and/or head and neck radiation as well as mIAS could thus lead to novel approaches for risk prediction of pain and responses to pain management. These strategic advances could substantively contribute to maintaining optimal cancer treatment protocol for each individual and enhancing his/her QOL during treatment.

## Clinical Assessment and Grading of mTOR Inhibitor–Associated Stomatitis

Although mIAS is recognized as a distinct clinical entity, clinical trials assessing mIAS have often utilized a variety of descriptive terms to identify this condition, including oral *mucositis* and *stomatitis*. The differential diagnosis of mIAS includes recurrent aphthous stomatitis, which may also occur during treatment but is unrelated to mTOR inhibition. Given the clinical similarities between mIAS and recurrent aphthous stomatitis 9, 10, the inability to differentiate between these two conditions is likely to result in overestimation of the true incidence of mIAS in these patients. Additionally, the incidence and natural history of mIAS have been reported by utilizing grading scales developed to assess conventional oral mucositis (e.g., World Health Organization, Oral Mucositis Assessment Scale, National Cancer Institute Common Terminology Criteria for Adverse Events [NCI CTCAE]) 9. However, applying criteria for oral mucositis caused by conventional chemotherapy and radiation therapy can lead to imprecise reporting of mIAS incidence, progression, and severity 9. For example, version 3.0 of the NCI CTCAE includes scales for subjective oral mucositis measures, including pain and ability to eat, and objective measures, including extent of erythema and ulcerations 9. Because most ulcers associated with mIAS are <0.5 cm in diameter, the majority of lesions would not be classified as grade 2 (patchy ulceration) by NCI CTCAE version 3.0. This approach would then potentially result in underreporting of mIAS severity 9. Version 4.0, in comparison, assesses symptoms and the functional effects of oral mucositis, focuses on the associated pain, and is more appropriately suited to grading mIAS 43.

Given these gaps in assessment technology, a scale specifically developed for evaluation and grading of mIAS in oncology patients was proposed by Boers‐Doets and Lalla 44. This innovative instrument includes measurements of subjective pain and objective measurements of lesion duration 44. This tool could lead to enhanced understanding of the functional and QOL impact of mIAS based on validation in future, larger studies. In this context, consideration of additional parameters, including the patient's ulcer frequency, size, and number and degree of difficulty while eating, could be valuable. Because the impact of pain on oral function is a consistent feature in mIAS, we advocate inclusion of a scale to evaluate a patient's nutritional intake as well.

## Impact of mTOR inhibitor–Associated Stomatitis on Therapy Administration

The effect of dose interruption or reduction of mTOR inhibitors in managing mIAS has been studied in several patient cohorts 9, 45. Martins and colleagues reported that dose reduction occurred in 19.2% of mTOR inhibitor‐treated patients, most frequently for thrombocytopenia (35.2%) and oral mucositis (27.3%) 9. In the BOLERO‐2 study, 24% of patients treated with everolimus plus exemestane required dose interruptions or adjustments for any‐grade mIAS 45. Most occurrences of mIAS were successfully managed with palliative interventions, including topical and systemic pain control; however, subsequent dose modifications were also documented 45. Despite adequate management of mIAS, it was the second most common toxicity that led to discontinuation of treatment (3% for everolimus plus exemestane vs. <1% for placebo plus exemestane) 45. However, in most patients with grade 3 mIAS in the everolimus plus exemestane arm, stomatitis resolved to grade ≤1 at 3.1 weeks and resolved completely in 82% of patients at 7.4 weeks 45.

## Preventing and Treating mTOR Inhibitor–Associated Stomatitis

Patient awareness, diligent monitoring, and timely management of mIAS are necessary to ensure that patients remain on mTOR inhibitor treatment 17, 18. As noted in Table 3, prophylactic measures to prevent mIAS have been proposed by expert panel groups 17, 46, 47. In comparison, study of the use of topical or systemic corticosteroids for mIAS treatment suggests their possible efficacy 14, 48, 49. A single‐center retrospective analysis of patients with cancer treated with the mTOR inhibitors everolimus or ridaforolimus determined that 87% of patients reported clinical improvement of mIAS (87%) after treatment with topical or systemic corticosteroids 48. This outcome is consistent with the report that topical clobetasol treatment reduced aphthous ulceration in renal transplant patients receiving sirolimus 50. In addition, the steroid mouth rinses and “magic” mouthwashes, often used for the palliative management of chemotherapy‐induced stomatitis, have been suggested for the prevention and treatment of mIAS 17, 46. A case report of a patient with mIAS for whom treatment included topical dexamethasone, intralesional injections of the corticosteroid triamcinolone, and magic mouthwash oral rinse demonstrated significant improvement in the signs and symptoms of mIAS lesions 47. However, the collective studies report an overall lack of effectiveness for managing mIAS with the prophylactic use of other oral rinses, such as antiseptic‐based 51 or sodium bicarbonate‐based mouthwashes 52.

**Table 3:** Suggested strategies to prevent or manage mammalian target of rapamycin inhibitor–associated stomatitis 17, 20, 46, 47

Prompt reporting
Educate patient on common signs and symptoms Educate patient to contact caregiver at first sign of mouth discomfort Educate patient to contact caregiver if lesions occur that interfere with eating and/or drinking
Basic oral care and oral hygiene
Instruct patient to: ○ Perform consistent, regular, and thorough brushing with a soft toothbrush; floss after each meal ○ Frequently rinse with bland rinses such as sterile water, normal saline, or sodium bicarbonate ○ Avoid alcohol‐containing rinses and toothpastes with sodium lauryl sulfate ○ Avoid alcohol‐ or peroxidase‐containing mouthwash products ○ Avoid acidic, spicy, hard, or crunchy foods that may injure the oral epithelium, and consume foods that are tepid rather than hot Consider use of oral moisturizers Emphasize need for regular dental examinations Treat anticipated infections (e.g., periodontal disease)
Assessment of other possible oral morbidities
Evaluate for herpetic, bacterial, and fungal infections Administer antimicrobials as appropriate

Pharmacologic treatment		
	Ingredients	Schedule
Topical local anesthetics^a^	Local anesthetics: lidocaine, benzocaine, butyl aminobenzoate, tetracaine hydrochloride	5 mL swish and expectorate as needed for pain, up to five times a day
Topical steroids	Dexamethasone, 0.5 mg/5 mL	5−10 mL swish and hold for 4–6 min, depending on patient tolerance, and expectorate; repeat three to four times daily
	Clobetasol gel, 0.05%	Apply to oral ulcers on a gauze pad; hold in place 5−10 min twice daily
	Prednisolone oral solution 15 mg/5 mL	5 mL swish and hold for 4–6 min, depending on patient tolerance, and expectorate; repeat three to four times daily
	Triamcinolone, 0.1% cream	Apply a thin film over the oral ulcer three times daily
Compounded steroid oral rinse	320 mL of diphenhydramine oral solution2 g of tetracycline powder 80 mg of hydrocortisone (four 20‐mg tablets crushed) 40 mL of nystatin suspension; dilute with water to 480 mL	10 mL solution four times daily (swish for 4–6 min, depending on patient tolerance, and expectorate)
Systemic steroids	Prednisone 5 mg	5 mg, one to two times daily (for severe resistant mIAS)

Dose adjustment of mTOR inhibitor (e.g., everolimus)^b^		
Grade	Symptoms	Everolimus dose modification
1	Minimal (normal diet)	No dose adjustment necessary
2	Symptomatic, but can eat and swallow modified diet	Temporary dose interruption until recovery to grade ≤1; reinitiate everolimus at the same doseIf stomatitis reoccurs at grade 2, interrupt dose until recovery to grade ≤1; reinitiate everolimus at a lower dose
3	Symptomatic and unable to adequately eat or hydrate orally	Temporary dose interruption until recovery to grade ≤1; reinitiate everolimus at a lower dose
4	Severe (symptoms are life threatening)	Discontinue everolimus and treat with medical therapy as indicated

mIAS, mTOR inhibitor–associated stomatitis; mTOR, mammalian target of rapamycin.

a Systemic pain medication may be needed with severe pain.

b As described in the package insert for everolimus 20.

The mIAS prevention and treatment strategies used in the BOLERO‐2 study and dose‐modification instructions highlighted in the everolimus package insert are also outlined in Table 3 20. Current management guidelines of oral mucosal injury focus on oral mucositis associated with chemotherapy or radiotherapy 8, 53, 54, 55. New guidelines specifically developed for the prevention and treatment of mIAS are needed.

## Future Directions

The current state‐of‐the‐science delineates at least three key areas for future research, as described in the sections below.

### Terminology

As mentioned, *mTOR inhibitor*–*associated stomatitis* 9, 10 has become the preferred descriptor over oral *mucositis* in order to differentiate the oral mucosal lesions associated with targeted therapies from the oral mucositis caused by conventional cancer therapy 7, 19. This revised terminology represents a valuable contribution to the literature in that it differentiates oral mucosal injury caused by conventional cancer treatment from oral mucosal injury caused by targeted cancer therapy. This terminology may further evolve in the future, when the pathobiology and clinical presentation of oral mucosal lesions caused by different types of molecularly targeted therapies become further defined. This evolution may result in elimination of the word “stomatitis,” which is broadly descriptive relative to inflammatory lesions of the oral cavity.

### Factors related to clinical risk prediction

Continued study of treatment‐related risk factors (e.g., drug, dosing schedule, administration route, concomitant therapies) and patient‐related risk factors (e.g., age, gender, body mass index, oral [including periodontal] health, hepatic or renal function, drug metabolism pharmacogenetics) could provide important insights into novel mechanistic pathways and associated targeted clinical management for mIAS. As previously noted, studying putative etiologies of recurrent aphthous ulceration, such as oral microbiota and genetic governance may be useful in assessing the risk for development of mIAS.

Recent advances in the computational molecular modeling of oral mucositis risk in the context of conventional high‐dose cancer therapies may provide novel insights into clinical risk prediction for mIAS as well 56, 57, 58. For example, single‐nucleotide polymorphisms (SNPs) derived from salivary DNA in patients undergoing hematopoietic stem cell transplants were assessed as predictors for risk of severe oral mucositis 59. An 82‐SNP Bayesian network related to risk was identified that had >99% cross‐validation accuracy and a predictive accuracy of 81% 59. It is interesting to consider whether such pharmacogenomic studies could also identify genetic predictors of mIAS risk. Although aphthous‐like ulcerations primarily affect nonkeratinized oral mucosal tissues 7, future studies into the pathobiology and genetic factors involved in mIAS should also assess nonoral mucosal tissue and other nonoral tissues, such as skin and nails, for potential associations.

### Prevention and treatment

#### Prevention

The everolimus package insert currently recommends that patients receiving everolimus for treatment of subependymal giant cell astrocytoma undergo measurement of an everolimus trough level 2 weeks after beginning therapy 20. In these patients, the dose is adjusted to attain trough concentrations of 5–15 ng/mL 20, an amount that may help prevent mIAS. However, it is currently unknown whether targeting the same everolimus trough concentration in patients with metastatic breast or renal cancer would reduce the incidence of mIAS.

#### Treatment

Ongoing studies of steroid mouth rinses are promising in that their results may contribute to new strategies for the reduction in clinical expression of mIAS. To this end, two trials (NCT02069093 and NCT02229136) in patients with metastatic breast cancer utilizing dexamethasone or prednisolone mouth rinses or compounded oral steroid rinse to reduce the incidence of or prevent mIAS are currently ongoing 60, 61 (Table 2). These trials are evaluating whether steroid mouth rinses can prevent and/or treat early mIAS, thereby decreasing the incidence of this toxicity and increasing the therapeutic index of mTOR inhibitor therapy.

Such studies of oral topical steroids are important because pharmacokinetic interactions between everolimus and a systemic steroid such as dexamethasone as mediated through the CYP3A4 metabolic pathway could lead to a reduction in the efficacy of everolimus. Topical oral dosing of the steroid minimizes this risk. However, prolonged corticosteroid exposure in the setting of chronic or severe mIAS with large areas of ulceration may result in adverse clinical sequelae secondary to elevated plasma steroid levels.

## Conclusions

mIAS occurs early during mTOR inhibitor treatment, is associated with significant morbidity, and can impair delivery of therapy. This can include women with invasive breast cancer who are receiving an mTOR inhibitor as a component of their treatment regimen. Prophylactic strategies, including oral hygiene and avoiding injury to the epithelium of the oral cavity, are recommended. The promising prevention and treatment strategies that are being evaluated in clinical trials are based, in part, on the observation of the clinical similarity and response to therapy of mIAS to recurrent aphthous ulceration. However, mIAS continues to be a clinically consequential toxicity in many patients. New research directed to the oral pain associated with mIAS could strategically enhance the clinical management of these patients, including preserving optimal treatment regimens while enhancing the patient's QOL.

## Conflict of Interest

D. E. Peterson has had a consultant/advisory role with Amgen, Cellceutix, Novartis, and Supportive Therapeutics. J. A. O'Shaughnessy has had a consultant/advisory role with Novartis. H. S. Rugo has received research funding from Novartis that was paid to the University of California Board of Regents. C. Campbell‐Baird has been remunerated by Amgen and Novartis for nursing education. J. Glaspy, M. M. Schubert, S. Elad, B. L. Schmidt, V. Seery, and C. T. Viet have no conflicts of interest to disclose. J. Divers has had a consultant/advisory role with Texas Oncology–Baylor Charles A. Sammons Cancer Center. J. Hronek has a consultant/advisory relationship with Genentech, Pfizer, and Merck. T. F. Meiller has had a consultant/advisory role with Novartis.

## References

[1] Sonis, S. T. 2011 Oral mucositis. Anticancer Drugs 22:607–612. 21709615 10.1097/CAD.0b013e3283462086

[2] Sonis, S. T. 2013 Oral mucositis in head and neck cancer: risk, biology, and management. Am. Soc. Clin. Oncol. Educ. Book. doi: 10.1200/EdBook_AM.2013.33.e236. 10.14694/EdBook_AM.2013.33.e236 23714511

[3] Epstein, J. B. , J. Thariat , R. J. Bensadoun , A. Barasch , B. A. Murphy , L. Kolnick , et al. 2012 Oral complications of cancer and cancer therapy: from cancer treatment to survivorship. CA Cancer J. Clin. 62:400–422. 22972543 10.3322/caac.21157

[4] Barasch, A. , and J. B. Epstein . 2011 Management of cancer therapy‐induced oral mucositis. Dermatol. Ther. 24:424–431. 21910800 10.1111/j.1529-8019.2011.01434.x

[5] Cheng, K. K. , V. Lee , C. H. Li , W. Goggins , D. R. Thompson , H. L. Yuen , et al. 2011 Incidence and risk factors of oral mucositis in paediatric and adolescent patients undergoing chemotherapy. Oral Oncol. 47:153–162. 21220206 10.1016/j.oraloncology.2010.11.019

[6] Peterson, D. E. , D. M. Keefe , and S. T. Sonis . 2012 New frontiers in mucositis. Am. Soc. Clin. Oncol. Educ. Book 2012:545–551. 24451793 10.14694/EdBook_AM.2012.32.46

[7] Boers‐Doets, C. B. , J. E. Raber‐Durlacher , N. S. Treister , J. B. Epstein , A. B. Arends , D. R. Wiersma , et al. 2013 Mammalian target of rapamycin inhibitor‐associated stomatitis. Future Oncol. 9:1883–1892. 24295418 10.2217/fon.13.141

[8] Peterson, D. E. , R. J. Bensadoun , C. Boers‐Doets , J. Herrstedt ; and ESMO Guidelines Committee . 2015 Management of oral and gastrointestinal mucosal injury ESMO clinical practice guidelines for diagnosis, treatment, and follow‐up. Ann. Oncol. 26(Suppl 5):v139–v151. 26142468 10.1093/annonc/mdv202

[9] Martins, F. , M. A. de Oliveira , Q. Wang , S. Sonis , M. Gallottini , S. George , et al. 2013 A review of oral toxicity associated with mTOR inhibitor therapy in cancer patients. Oral Oncol. 49:293–298. 23312237 10.1016/j.oraloncology.2012.11.008

[10] Sonis, S. , N. Treister , S. Chawla , G. Demetri , and F. Haluska . 2010 Preliminary characterization of oral lesions associated with inhibitors of mammalian target of rapamycin in cancer patients. Cancer 116:210–215. 19862817 10.1002/cncr.24696

[11] Sankhala, K. , A. Mita , K. Kelly , D. Mahalingam , F. Giles , and M. Mita . 2009 The emerging safety profile of mTOR inhibitors, a novel class of anticancer agents. Target Oncol. 4:135–142. 19381454 10.1007/s11523-009-0107-z

[12] Chavez‐MacGregor, M. , and A. M. Gonzalez‐Angulo . 2012 Everolimus in the treatment of hormone receptor‐positive breast cancer. Expert Opin. Investig. Drugs 21:1835–1843. 10.1517/13543784.2012.726218 22994502

[13] Suzuki, A. , R. Kobayashi , S. Okayasu , B. Kuze , M. Aoki , K. Mizuta , et al. 2014 Pharmacotherapy for adverse events reduces the length of hospital stay in patients admitted to otolaryngology ward: a single arm intervention study. PLoS ONE 9:e115879. 25549093 10.1371/journal.pone.0115879 PMC4280125

[14] Villa, A. , A. Aboalela , K. A. Luskin , C. S. Cutler , S. T. Sonis , S. B. Woo , et al. 2015 Mammalian target of rapamycin inhibitor‐associated stomatitis in hematopoietic stem cell transplantation patients receiving sirolimus prophylaxis for graft‐versus‐host disease. Biol. Blood Marrow Transplant. 21:503–508. 25482865 10.1016/j.bbmt.2014.11.680

[15] Abdel‐Rahman, O. , and M. Fouad . 2014 Risk of mucocutaneous toxicities in patients with solid tumors treated with sorafenib: an updated systematic review and meta‐analysis. Expert Rev. Anticancer Ther. 14:751–760. 24666215 10.1586/14737140.2014.894465

[16] Yardley, D. A. 2014 Adverse event management of mtor inhibitors during treatment of hormone receptor‐positive advanced breast cancer: considerations for oncologists. Clin. Breast Cancer 14:297–308. 25065566 10.1016/j.clbc.2014.03.002

[17] Divers, J. , and J. O'Shaughnessy . 2015 Stomatitis associated with use of mtor inhibitors: implications for patients with invasive breast cancer. Clin. J. Oncol. Nurs. 19:468–474. 26207713 10.1188/15.CJON.468-474

[18] Meiller, T. F. , S. Varlotta , and D. Weikel . 2015 Recognition and management of oral mucosal injury caused by mammalian target of rapamycin inhibitors: a case series. Case Rep. Oncol. 8:369–377. 26464573 10.1159/000438747 PMC4592504

[19] Watters, A. L. , J. B. Epstein , and M. Agulnik . 2011 Oral complications of targeted cancer therapies: a narrative literature review. Oral Oncol. 47:441–448. 21514211 10.1016/j.oraloncology.2011.03.028

[20] Novartis Pharmaceuticals Corporation . 2014 Afinitor (everolimus tablets for oral administration). Afinitor Disperz (everolimus tablets for oral suspension) [package insert]. Novartis Pharmaceuticals Corporation, East Hanover, NJ.

[21] Pfizer . 2012 Torisel Kit (temsirolimus) injection, for intravenous infusion only [package insert]. Pfizer, Philadelphia, PA.

[22] Morgenstern, D. A. , M. Marzouki , U. Bartels , M. S. Irwin , G. L. Sholler , J. Gammon , et al. 2014 Phase I study of vinblastine and sirolimus in pediatric patients with recurrent or refractory solid tumors. Pediatr. Blood Cancer 61:128–133. 23956145 10.1002/pbc.24656

[23] Al‐Dasooqi, N. , S. T. Sonis , J. M. Bowen , E. Bateman , N. Blijlevens , R. J. Gibson , et al. 2013 Emerging evidence on the pathobiology of mucositis. Support. Care Cancer 21:2075–2083. 23604521 10.1007/s00520-013-1810-y

[24] Sonis, S. T. 1998 Mucositis as a biological process: a new hypothesis for the development of chemotherapy‐induced stomatotoxicity. Oral Oncol. 34:39–43. 9659518 10.1016/s1368-8375(97)00053-5

[25] Talwar, S. , R. House , S. Sundaramurthy , S. Balasubramanian , H. Yu , and V. Palanisamy . 2014 Inhibition of caspases protects mice from radiation induced oral mucositis and abolishes the cleavage of RNA binding protein HuR. J. Biol. Chem. 289:3487–3500. 24362034 10.1074/jbc.M113.504951 PMC3916550

[26] Wardill, H. R. , J. M. Bowen , N. Al‐Dasooqi , M. Sultani , E. Bateman , R. Stansborough , et al. 2014 Irinotecan disrupts tight junction proteins within the gut: implications for chemotherapy‐induced gut toxicity. Cancer Biol. Ther. 15:236–244. 24316664 10.4161/cbt.27222 PMC3928140

[27] Mougeot, J. L. , F. K. Mougeot , D. E. Peterson , R. J. Padilla , M. T. Brennan , and P. B. Lockhart . 2013 Use of archived biopsy specimens to study gene expression in oral mucosa from chemotherapy‐treated cancer patients. Oral Surg. Oral Med. Oral Pathol. Oral Radiol. 115:630–637. 23541936 10.1016/j.oooo.2013.01.003

[28] Katholnig, K. , M. Linke , H. Pham , M. Hengstschlager , and T. Weichhart . 2013 Immune responses of macrophages and dendritic cells regulated by mTOR signalling. Biochem. Soc. Trans. 41:927–933. 23863158 10.1042/BST20130032 PMC6322655

[29] Lewkowicz, N. , P. Lewkowicz , K. Dzitko , B. Kur , M. Tarkowski , A. Kurnatowska , et al. 2008 Dysfunction of CD4+ CD25high T regulatory cells in patients with recurrent aphthous stomatitis. J. Oral Pathol. Med. 37:454–461. 18318707 10.1111/j.1600-0714.2008.00661.x

[30] Bankvall, M. , F. Sjoberg , G. Gale , A. Wold , M. Jontell , and S. Ostman . 2014 The oral microbiota of patients with recurrent aphthous stomatitis. J. Oral Microbiol. 6:25739. 25626771 10.3402/jom.v6.25739 PMC4221501

[31] Hijazi, K. , T. Lowe , C. Meharg , S. H. Berry , J. Foley , and G. L. Hold . 2015 Mucosal microbiome in patients with recurrent aphthous stomatitis. J. Dent. Res. 94:87S–94S. 25540188 10.1177/0022034514565458 PMC4541092

[32] Koybasi, S. , A. H. Parlak , E. Serin , F. Yilmaz , and D. Serin . 2006 Recurrent aphthous stomatitis: investigation of possible etiologic factors. Am. J. Otolaryngol. 27:229–232. 16798397 10.1016/j.amjoto.2005.09.022

[33] Chen, H. , Q. Sui , Y. Chen , L. Ge , and M. Lin . 2015 Impact of haematologic deficiencies on recurrent aphthous ulceration: a meta‐analysis. Br. Dent. J. 218:E8. 25720915 10.1038/sj.bdj.2015.100

[34] Kozlak, S. T. , S. J. Walsh , and R. V. Lalla . 2010 Reduced dietary intake of vitamin B12 and folate in patients with recurrent aphthous stomatitis. J. Oral Pathol. Med. 39:420–423. 20141576 10.1111/j.1600-0714.2009.00867.x PMC3323114

[35] Slebioda, Z. , E. Szponar , and A. Kowalska . 2013 Recurrent aphthous stomatitis: genetic aspects of etiology. Postepy. Dermatol. Alergol. 30:96–102. 24278055 10.5114/pdia.2013.34158 PMC3834687

[36] Karasneh, J. , M. Bani‐Hani , A. Alkhateeb , A. Hassan , F. Alzoubi , and M. Thornhill . 2014 TLR2, TLR4 and CD86 gene polymorphisms in recurrent aphthous stomatitis. J. Oral Pathol. Med. 44:857–863. 25482673 10.1111/jop.12298

[37] Sessa, C. , D. Tosi , L. Vigano , J. Albanell , D. Hess , M. Maur , et al. 2010 Phase Ib study of weekly mammalian target of rapamycin inhibitor ridaforolimus (AP23573; MK‐8669) with weekly paclitaxel. Ann. Oncol. 21:1315–1322. 19901013 10.1093/annonc/mdp504

[38] Scully, C. 2006 Clinical practice. Aphthous ulceration. N. Engl. J. Med. 355:165–172. 16837680 10.1056/NEJMcp054630

[39] Elting, L. S. , D. M. Keefe , S. T. Sonis , A. S. Garden , F. K. Spijkervet , A. Barasch , et al. 2008 Patient‐reported measurements of oral mucositis in head and neck cancer patients treated with radiotherapy with or without chemotherapy: demonstration of increased frequency, severity, resistance to palliation, and impact on quality of life. Cancer 113:2704–2713. 18973181 10.1002/cncr.23898

[40] Viet, C. T. , P. M. Corby , A. Akinwande , and B. L. Schmidt . 2014 Review of preclinical studies on treatment of mucositis and associated pain. J. Dent. Res. 93:868–875. 24943201 10.1177/0022034514540174 PMC4213248

[41] McMahon, S. , M. Koltzenburg , I. Tracey , and D. C. Turk . 2011 Wall & Melzack's textbook of pain, 6th ed. WB Saunders Co, St Louis, MO.

[42] Sonis, S. T. 2004 The pathobiology of mucositis. Nat. Rev. Cancer 4:277–284. 15057287 10.1038/nrc1318

[43] US Department of Health and Human Services . 2010 Common terminology criteria for adverse events (CTCAE), v4.03. Available at: http://evs.nci.nih.gov/ftp1/CTCAE/CTCAE_4.03_2010-06-14_QuickReference_5x7.pdf. June 14, 2010. (accessed 19 June 2015).

[44] Boers‐Doets, C. , and R. V. Lalla . 2013 The mIAS scale: a scale to measure mTOR inhibitor‐associated stomatitis. Supp. Care Cancer 21(Suppl. 1):1–6 Abstract MASCC‐0396.

[45] Rugo, H. S. , K. I. Pritchard , M. Gnant , S. Noguchi , M. Piccart , G. Hortobagyi , et al. 2014 Incidence and time course of everolimus‐related adverse events in postmenopausal women with hormone receptor‐positive advanced breast cancer: insights from BOLERO‐2. Ann. Oncol. 25:808–815. 24615500 10.1093/annonc/mdu009 PMC3969554

[46] Porta, C. , S. Ostanto , A. Ravaud , M. A. Climent , U. Vaishampayan , D. A. White , et al. 2011 Management of adverse events associated with the use of everolimus in patients with advanced renal cell carcinoma. Eur. J. Cancer 47:1287–1298. 21481584 10.1016/j.ejca.2011.02.014

[47] Pilotte, A. P. , M. B. Hohos , K. M. Polson , T. M. Huftalen , and N. Treister . 2011 Managing stomatitis in patients treated with mammalian target of rapamycin inhibitors. Clin. J. Oncol. Nurs. 15:E83–E89. 21951751 10.1188/11.CJON.E83-E89

[48] de Oliveira, M. A. , E. M. F. Martins , Q. Wang , S. Sonis , G. Demetri , S. George , et al. 2011 Clinical presentation and management of mTOR inhibitor‐associated stomatitis. Oral Oncol. 47:998–1003. 21890398 10.1016/j.oraloncology.2011.08.009

[49] Kalogirou, E. M. , K. I. Tosios , E. P. Piperi , and A. Sklavounou . 2015 mTOR inhibitor‐associated stomatitis (mIAS) in three patients with cancer treated with everolimus. Oral Surg. Oral Med. Oral Pathol. Oral Radiol. 119:e13–e19. 25442249 10.1016/j.oooo.2014.08.023

[50] Chuang, P. , and A. J. Langone . 2007 Clobetasol ameliorates aphthous ulceration in renal transplant patients on sirolimus. Am. J. Transplant. 7:714–717. 17250555 10.1111/j.1600-6143.2006.01678.x

[51] Raymond, E. , J. Alexandre , S. Faivre , K. Vera , E. Materman , J. Boni , et al. 2004 Safety and pharmacokinetics of escalated doses of weekly intravenous infusion of CCI‐779, a novel mTOR inhibitor, in patients with cancer. J. Clin. Oncol. 22:2336–2347. 15136596 10.1200/JCO.2004.08.116

[52] Ferte, C. , A. Paci , M. Zizi , D. B. Gonzales , A. Goubar , C. Gomez‐Roca , et al. 2011 Natural history, management and pharmacokinetics of everolimus‐induced‐oral ulcers: insights into compliance issues. Eur. J. Cancer 47:2249–2255. 21489779 10.1016/j.ejca.2011.03.017

[53] Rubenstein, E. B. , D. E. Peterson , M. Schubert , D. Keefe , D. McGuire , J. Epstein , et al. 2004 Clinical practice guidelines for the prevention and treatment of cancer therapy‐induced oral and gastrointestinal mucositis. Cancer 100:2026–2046. 15108223 10.1002/cncr.20163

[54] Keefe, D. M. , M. M. Schubert , L. S. Elting , S. T. Sonis , J. B. Epstein , J. E. Raber‐Durlacher , et al. 2007 Updated clinical practice guidelines for the prevention and treatment of mucositis. Cancer 109:820–831. 17236223 10.1002/cncr.22484

[55] Lalla, R. V. 2013 The MASCC/ISOO mucositis guidelines update: introduction to the first set of articles. Support. Care Cancer 21:301–302. 23161340 10.1007/s00520-012-1660-z

[56] Peterson, D. , R. Srivastava , and R. Lalla . 2015 Oral mucosal injury in oncology patients: perspectives on maturation of a field. Oral Dis. 21:133–141. 24131518 10.1111/odi.12167

[57] Jensen, S. B. , and D. E. Peterson . 2014 Oral mucosal injury caused by cancer therapies: current management and new frontiers in research. J. Oral Pathol. Med. 43:81–90. 24261541 10.1111/jop.12135

[58] Hahn, T. , E. Zhelnova , L. Sucheston , I. Demidova , V. Savchenko , M. Battiwalla , et al. 2010 A deletion polymorphism in glutathione‐S‐transferase mu (GSTM1) and/or theta (GSTT1) is associated with an increased risk of toxicity after autologous blood and marrow transplantation. Biol. Blood Marrow Transplant. 16:801–808. 20074657 10.1016/j.bbmt.2010.01.001

[59] Sonis, S. T. , J. H. Antin , M. W. Tedaldi , and G. Alterovitz . 2013 SNP‐based Bayesian networks can predict oral mucositis risk in autologous stem cell transplant recipients. Oral Dis. 19:721–727. 23809011 10.1111/odi.12146

[60] US Oncology Research . 2000 Miracle mouthwash plus hydrocortisone vs prednisolone mouth rinse for mouth sores caused by everolimus. In: ClinicalTrials.gov. National Library of Medicine (US), Bethesda (MD) Available at: https://clinicaltrials.gov/ct2/show/NCT02229136 NLM Identifier: NCT02229136 (accessed 21 September 2015).

[61] Novartis Pharmaceuticals . 2000 Open‐label, phase II study of stomatitis prevention with a steroid‐based mouthwash in post‐menopausal women with ER+, HER2‐ metastatic or locally advanced breast cancer. In: ClinicalTrials.gov. National Library of Medicine (US), Bethesda (MD) Available at: https://clinicaltrials.gov/ct2/show/NCT02069093 NLM Identifier: NCT02069093 (accessed 21 September 2015).

[62] Sonis, S. T. 2009 Mucositis: the impact, biology and therapeutic opportunities of oral mucositis. Oral Oncol. 45:1015–1020. 19828360 10.1016/j.oraloncology.2009.08.006

